# Near-Lifespan Tracking of Cerebral Microvascular Degeneration in Aging to Alzheimer’s Continuum

**DOI:** 10.20900/agmr20220003

**Published:** 2022-03-29

**Authors:** Jonghwan Lee

**Affiliations:** 1 Center for Biomedical Engineering, School of Engineering, Brown University, Providence, RI 02912, USA; 2 Carney Institute for Brain Science, Brown University, Providence, RI 02912, USA

**Keywords:** Alzheimer’s disease, vascular factor, microvascular degeneration, longitudinal tracking, integrative pathophysiology

## Abstract

Alzheimer’s disease (AD) is a progressive neurodegenerative disorder affecting millions of people worldwide and is currently incurable. As the population ages, AD and related dementia are becoming the biggest epidemic in medical history: the number of people aged 65 and older with AD is projected to increase between two- and three-fold by 2050. Imaging and biomarker studies suggest that the pathophysiological processes of AD begin more than a decade before the diagnosis of dementia, opening the possibility of early, preemptive prediction. For accurate prediction, it is important although challenging to fully understand how multiple etiologies and age-related prodromal processes contribute to the onset of Alzheimer’s continuum, across a long period comparable to the lifespan. Addressing this challenge was one of the overarching transformative concepts at the 2015 AD Research Summit, “to develop new programs on *systems biology and integrative physiology* to gain a deeper understanding of the complex biology of the disease.”

Among other factors, cerebral microvascular degeneration (CMD) may play a key role in the onset and development of Alzheimer’s continuum, potentially prior to, along with, or independently of the beta-amyloid (Aβ) accumulation. Despite its importance for early detection and as a therapeutic target for early intervention, it is unknown whether CMD is a causal factor for AD pathogenesis or an early consequence of multifactorial conditions that lead to AD at a later stage. Here, this *Viewpoint* suggests that we should fill two critical knowledge gaps: (1) Temporal relationships between various CMDs and other key factors before/during/after the onset of Alzheimer’s continuum have not been established; (2) Little integrative study down to the capillary vessel level has been conducted on how individual defects in various microvascular structural and flow properties distinctly correlate with and/or contribute to neuronal degeneration. As the first step toward filling these gaps, I propose utilizing recent advances in microscopic imaging and image analysis techniques to longitudinally track a comprehensive set of CMDs over the lifespan in model animals, along with Aβ, tau, neuronal degeneration, and cognitive impairment when possible.

## MICROVASCULAR DYSFUNCTION CAN CAUSE NEURONAL DEGENERATION

Neurons depend on blood vessels for oxygen and nutrients, as well as for removal of carbon dioxide and other potentially toxic metabolites. For normal functioning of neuronal circuits, cerebral blood flow (CBF) should be well maintained during the resting state and properly regulated in response to neural activation to meet the metabolic demand of neurons, location by location, moment by moment [1]. At the microvascular scale, cortical capillary blood vessels play an important role in this CBF supply and regulation [2], and thus in normal functioning of neuronal circuits.

Among various pathways by which a microvascular deficit causes neuronal degeneration, major players include hypoperfusion, dysregulated functional hyperemia, and reduced clearance [3] (See Ref. [4] for a comprehensive list of related vascular pathways). First, severe hypoperfusion leading to hypoxia causes an array of detrimental effects on neurons [5]; even mild hypoperfusion decreases protein synthesis in neurons and eventually leads to a cascade of neuronal loss [6]. Hypoperfusion also alters electrolyte balances and water gradients, leading to edema and white matter damage, and interestingly, facilitates the accumulation of Aβ plaques [7–10]. At the microvascular scale, capillary vessel flow patterns are directly related to the extraction efficacy of oxygen in the brain parenchyma [11,12]; a deficit in the flow pattern like an increased heterogeneity may lead to neurodegeneration even in the absence of artery-level hypoperfusion [13]. Second, dysregulated functional hyperemia or neurovascular coupling is also believed to lead to or be associated with neuronal damage in many disorders, some of which may occur independently of the resting-state hypoperfusion [14,15]. Third, influx and efflux of interstitial fluid (ISF) and cerebrospinal fluid (CSF) affect brain transfer of intravascular substances. Dysfunction in two related pathways, the intramural peri-arterial drainage pathway of ISF and the glymphatic system pathway of CSF, may contribute to neuronal degeneration via ineffective waste clearance [16].

Blood-brain barrier (BBB), although not within the vascular system, also plays a key role in neuronal degeneration when it dysfunctions; thus, this *Viewpoint* includes BBB dysfunction to a broad definition of cerebral microvascular degeneration (CMD). BBB breakdown leads to leaking and/or formation of components toxic to neurons, including thrombin and reactive oxygen species [17,18], and results in hypoperfusion through albumin-promoted vasogenic edema [19].

## VARIOUS CEREBRAL MICROVASCULAR DEGENERATIONS ARE OBSERVED IN ALZHEIMER’S DISEASE

Hypoperfusion is observed near the onset of Alzheimer’s continuum in patients and animal models. The degree of hypoperfusion differs by brain regions and measurement techniques, but many imaging studies consistently show significant hypoperfusion in the hippocampus and parietal and temporal cortices [20–30]. A population-based study found that cerebral hypoperfusion is associated with accelerated cognitive decline and an increased risk of dementia [31,32]. Studies using transgenic mice observed hypoperfusion even at young ages when there are no amyloid plaques [33], as well as a potent link between cerebral hypoperfusion and parenchymal Aβ pathology [34]. At the microvascular scale, both structural defects and functional deficits are observed in AD, such as more string vessels, reduced capillary density, increased tortuosity, thickened and irregular basement membranes, increased BBB permeability, and increased capillary transit time heterogeneity [35–39]. A recent study using in-vivo two-photon fluorescence microscopy found that AD mice exhibit stalled blood flow in more cortical capillaries than wild-type mice [40].

In addition to these deficits in vascular structures and resting-state CBF, dysregulations in functional hyperemia or neurovascular coupling are observed in AD patients and animal models. Imaging studies revealed such dysregulations during memory-related tasks [41,42], verbal tasks [43,44], and in response to visual stimuli [45,46]. At the microvascular scale, early homogenization of capillary blood flow in response to functional activation, which we demonstrated in wild-type animals [47], is absent in 18-month-old AD mice [48].

These CMDs likely interact with Aβ accumulation in a complicated manner, as Aβ often deposits on the vessel wall, leading to vascular dysfunction, while the hypoperfusion accelerates Aβ accumulation. And the BBB is an important player in these complicated interactions. For example, a magnetic resonance imaging study found that water exchange rate across the BBB is associated with cerebrospinal fluid (CSF) Aβ_42_ in healthy older adults [49]. This feed-forward interaction makes it particularly difficult to clarify the mechanistic roles of CMD in AD. But a human study published in 2020 reported a very interesting finding [50]. Carriers of APOE4, a major risk gene in sporadic AD, exhibited higher BBB breakdown in the hippocampus and medial temporal lobe when their cognitive impairment was either absent or very mild. More importantly, the observed BBB breakdown was independent of CSF levels of Aβ and Tau. This independence suggests that early CMD may serve as an initial pathogenesis in Alzheimer’s continuum separately from the Aβ pathogenesis, although two factors may feed-forward each other in a later stage of the disease.

## TWO CRITICAL KNOWLEDGE GAPS REGARDING CMD IN ALZHEIMER’S CONTINUUM

Despite these increasing pieces of evidence, temporal relationships between CMD and other key factors in the onset and development of Alzheimer’s continuum are not clear. This knowledge gap was pinpointed by a comprehensive review [3]: “The *time course* of these vascular alterations and how they relate to dementia and Alzheimer’s disease pathology remain unclear, as no protocol that allows the development of the diverse brain vascular pathology to be scored, and hence *to be tracked with ageing*, has so far been developed and widely validated.” Since then, a multifactorial data-driven analysis led to a tentative temporal ordering of various factors in the progression of late-onset AD [51]. This study suggested that vascular alterations may occur earlier than the other factors, but these time courses have not been tracked in real experiments with aging.

Second, each of various CMDs will have a different degree of contribution to, or correlation with, neuronal degeneration. However, most prior research has investigated only a few structural defects or flow deficits at a time, making it difficult to compare the degrees of contribution quantitatively across different studies. A more rigorous approach will be to track and compare a comprehensive set of CMDs at once within the same subject (i.e., in a longitudinal manner). More importantly, few studies investigated CMDs in multiple scales at once, from pial vessels to individual capillaries.

## CONCLUSIONS

The major challenge in filling the knowledge gap is that Alzheimer’s continuum develops very slowly and thus it is nearly impossible to longitudinally track CMD in humans for a long period comparable to a fraction of the lifespan. Such longitudinal tracking has been challenging even in model animals with much shorter lifespans. Here this *Viewpoint* suggests that recent advances in microscopic rodent brain imaging and image analysis techniques may enable it (see Ref. [52] for example), and the AD research community should utilize the advanced techniques in model animals to fill the critical knowledge gaps. Findings from such studies will provide unprecedented insight into the mechanistic roles of CMD in Alzheimer’s continuum. They will in turn facilitate translational/clinical research for CMD-based early detection and treatment of the disease that is becoming the biggest epidemic.
